# Delayed Diagnosis of an Invisible Seizure: Cefepime-Induced Non-convulsive Status Epilepticus

**DOI:** 10.7759/cureus.46810

**Published:** 2023-10-10

**Authors:** Saint-Martin Allihien, Sammudeen Ibrahim, Prabina Basnet, Kashish Palla, James Appiah-Pippim

**Affiliations:** 1 Internal Medicine, Piedmont Athens Regional Medical Center, Athens, USA; 2 Graduate Medical Education, Piedmont Athens Regional Medical Center, Athens, USA; 3 Medicine, Lambert High School, Suwanee, USA; 4 Pulmonary and Critical Care Medicine, Piedmont Athens Regional Medical Center, Athens, USA

**Keywords:** myoclonic twitches, pseudomonas aeruginosa, renal dosing, acute kidney injury(aki)), electroencephalography (eeg), cefepime-induced seizures, non-convulsive status epilepticus

## Abstract

Cefepime-induced non-convulsive status epilepticus (NCSE) is a recognized adverse event of cefepime. Risk factors for this adverse event include older age, underlying renal dysfunction, previous brain injury, diabetes, and severe infection. We present a case of a 79-year-old woman with no prior seizure history, who was admitted for Pseudomonas aeruginosa surgical wound infection for which she was on cefepime. She developed acute encephalopathy with associated, occasional, right-sided myoclonic facial twitches 11 days into her admission. Electroencephalogram (EEG) confirmed NCSE as evident by epileptiform activity described as generalized periodic discharges with predominantly triphasic morphology. Cefepime was substituted with piperacillin-tazobactam> 24 hours after symptom onset. NCSE completely resolved two days after the discontinuation of cefepime. This case highlights the fact that NCSE can occur even when precautions such as renal dosing of cefepime are observed. Clinicians need to have a high index of suspicion for the condition when taking care of at-risk patients on cefepime, as delayed diagnosis correlates with potentially fatal outcomes.

## Introduction

Status epilepticus has been previously defined as continuous seizure activity lasting greater than 30 minutes. This definition has however been revised to describe continuous clinical/electrographic seizure activity lasting more than five minutes or recurrent seizure activity without recovery in between seizures during this five-minute period. In the absence of tonic-clonic movement, electrographic status is termed non-convulsive status epilepticus (NCSE) [1,2]. NCSE is a fatal condition with an in-hospital mortality ranging from 18% to 52% and a reported 30-day mortality rate as high as 60% [2].

Cefepime is one of the few cephalosporin antibiotics with broad activity against gram-positive and gram-negative aerobes, including Pseudomonas aeruginosa [3]. For this reason, it is commonly used in many clinical settings. However, its use has been associated with neurologic side effects, including acute encephalopathy, myoclonus, and NCSE [4-7]. To avoid this adverse effect, the Food and Drugs Authority (FDA) advised healthcare practitioners, in a communique released in 2012, to renally dose cefepime [8]. Nonetheless, cefepime-induced NCSE remains less well-understood by many clinicians with a potential for misdiagnosis and delayed diagnosis. We present a case of delayed diagnosis of cefepime-induced NCSE in a 79-year-old female who developed an adverse event even though she was on renally dosed cefepime.

## Case presentation

**Figure 1:**
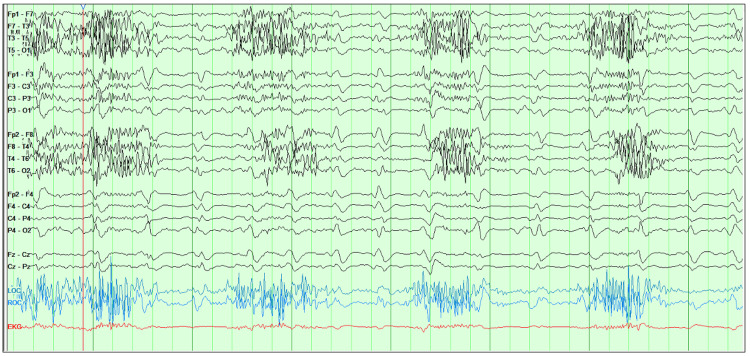
EEG prior to the discontinuation of cefepime showing generalized periodic discharges with predominantly triphasic morphology

A 79-year-old woman with no prior history of seizures had lumbar laminectomies and bone biopsy for osteomyelitis and discitis and had been on cefadroxil and doxycycline. About three weeks post-surgery, the patient was readmitted for surgical wound infection and was initially started on renally dosed cefepime and vancomycin in the setting of acute kidney injury. She was later continued on cefepime alone, as the wound culture grew Pseudomonas aeruginosa. She developed acute encephalopathy on day 11 of her admission, which was characterized by the patient being nonverbal. The Glasgow Coma Scale score was 8 and she had occasional right-sided myoclonic twitches of the face. Head computed tomography scan and head magnetic resonance imaging were unremarkable for acute findings. She had no metabolic or electrolyte abnormalities that could explain her change in mental status. There was no indication of worsening surgical site infection, and repeat blood cultures and urine cultures were negative. About 12 hours after the onset of symptoms, a continuous electroencephalogram (EEG) was obtained and showed epileptiform activity described as generalized periodic discharges with predominantly triphasic morphology (Figure 1). There was however no associated tonic-clonic seizure activity.

**Figure 2:**
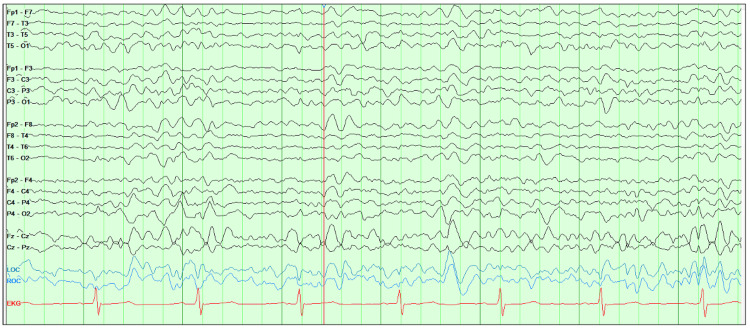
EEG two days after discontinuing cefepime, showing markedly improved EEG with resolution of generalized periodic epileptiform discharge pattern

The patient was started on levetiracetam and sodium valproate without resolution of electrographic seizure activity. She remained on cefepime for three more doses and for more than 24 hours following the onset of symptoms before it was finally substituted for piperacillin-tazobactam on suspicion that cefepime may be contributing to non-convulsive seizures. Two days after the discontinuation of cefepime, the symptoms resolved, with EEG showing resolution of epileptiform activity as shown in Figure 2.

## Discussion

Two distinct phenotypes of NCSE have been described. One phenotype is the “wondering confused patient”, which is often observed in patients presenting in the emergency department who often have a history of epileptic syndromes. The prognosis associated with this first type of NCSE is good. The second phenotype occurs in acutely ill patients with severely impaired mental status. This second category of patients may present with or without subtle motor movements, such as muscle twitches or tonic eye deviation, and is also known as “subtle status”. NCSE in this later population of patients often follows generalized convulsive status epilepticus and is usually observed in intensive care unit settings [2]. Epileptogenic EEG finding of generalized periodic discharges with predominantly triphasic morphology has been frequently associated with NCSE [9,10]. Our patient exhibited symptoms in keeping with the second phenotype of NCSE and had the typical EEG finding; however, she had no previous history of seizure disorder.

Cefepime, like other cephalosporins, has neurotoxic side effects that commonly manifest as reduced consciousness [11,12]. Cefepime-induced NCSE may present as acute encephalopathy (82%), but it can present as lethargy or coma (22%), speech disturbances (15%), myoclonus (13%), and hallucinations (6%) [13]. The neurotoxicity of cefepime is reported to be mediated by its affinity to gamma-aminobutyric acid (GABA)-A receptors of the central nervous system (CNS), which is stronger than that of many -lactams. GABA-mediated inhibitory response is thus reduced resulting in CNS excitation. Furthermore, cefepime’s lipophilic property enables it to achieve high concentrations in the CNS especially the brain [12].

A high serum concentration of cefepime is associated with an increased likelihood of neurotoxic adverse events, including NSCE. Although serum cefepime concentration is an important determinant of neurotoxicity, there is no consensus on what the appropriate serum threshold of the drug should be. While earlier reports suggested that a cefepime trough concentration of 22 mg/L may predict the likelihood of neurotoxicity, attempts to confirm such a report by Rhodes et al. revealed that this reported serum concentration had low precision as a predictive threshold [14,15]. A relatively recent retrospective study on the subject suggested a higher threshold plasma concentration of 35 mg/L beyond which there is an increased risk of neurotoxicity with cefepime [16]. This later result is congruent with the findings of a systematic review that reported that 76% of patients who experienced cefepime neurotoxicity had serum concentrations of 35 mg/dl [11].

NCSE is more common in patients with underlying renal dysfunction [8]. Since about 85% of cefepime is renally excreted, renal dysfunction can exponentially increase the half-life of the drug from 2 hours to 13 hours, thereby increasing the risk of toxicity [17]. A similar process is responsible for NCSE with other medications such as baclofen and tiagabine [18,19]. Renal dosing of cefepime is therefore critical in reducing the risk of NCSE in patients with underlying renal dysfunction. This, however, can prove challenging, especially in critically ill patients as renal function may be overestimated [20]. Abnormalities associated with renal dysfunction, such as proteinuria and hypoalbuminemia, may affect drug pharmacodynamics, resulting in a relatively larger pharmacologically active unbound fraction compared to the reported median unbound fraction of 61.4%. Such an environment increases the risk of CNS toxicity [21]. That notwithstanding, the neurotoxic effects of cefepime may still occur in patients with normal renal function or when the medication is renally dosed [22]. Other factors, such as inflammatory conditions, serum inorganic acids, severe infection, diabetes, and previous brain injury, may disrupt the blood brain barrier integrity, increasing CNS penetration of the drug with a resultant risk of NCSE [23,24]. The adverse effect is also more reported in patients who are 50 years and older [8].

In a systematic analysis by Payne et al., the median onset of NCSE in patients receiving cefepime was reported to be four days from initiation of therapy, with an interquartile range (IQR) of two to six days [12]. The Neurocritical Care Society recommends continuous EEG monitoring as part of the diagnostic workup of NCSE [2]. Treatment, like in many adverse drug events, includes discontinuation of the offending drug; this often results in complete resolution of symptoms after a median of two days. Antiseizure medicines may also be used. Given that the biochemical mechanism of cefepime-induced NCSE involves GABA-A receptor antagonism, GABA agonists such as benzodiazepines may play an important role in treatment. It is also reasonable to adjust the dosage of cefepime in patients with underlying kidney disease who develop NCSE [8,11,22]. Dialysis has been used to reduce serum cefepime concentration and effectively abort NCSE [4]. Early diagnosis and treatment of NCSE within 30 minutes of seizure onset is associated with 36% mortality compared to 75% mortality for patients diagnosed at >24 hours of seizure onset [2].

Our case is an example of an atypical presentation of a known adverse effect. Our patient experienced NCSE side effects of cefepime even though it was renally dosed. While the median time of onset of NCSE is four days (IQR 2-6) from the initiation of cefepime, our patient experienced the adverse effect 11 days after initiation of the medication. These, together with the fact that NCSE is not a condition that clinicians are familiar with, led to a delayed diagnosis 24 hours after the onset of her symptoms.

## Conclusions

Cefepime-induced NCSE is a potentially fatal adverse drug event. Early diagnosis and treatment are critical to improve prognosis and neurologic recovery. Therefore, clinicians need to have a high index of suspicion for patients on cefepime, particularly those with risk factors reported to be associated with the NCSE such as older age, underlying renal dysfunction, previous brain injury, diabetes, and severe infection. While we cannot overemphasize the importance of renally dosing cefepime in patients with underlying renal dysfunction, clinicians should be aware that this fatal adverse event can still occur in patients with normal baseline renal function.
